# Secondary Gamma-Delta T-Cell Lymphoma Not Otherwise Specified (NOS) From Chronic Immunosuppression

**DOI:** 10.7759/cureus.14808

**Published:** 2021-05-02

**Authors:** Madeleine E Turcotte, Amar H Kelkar, Joanna Chaffin, Nam H Dang

**Affiliations:** 1 Department of Internal Medicine, Vanderbilt University Medical Center, Nashville, USA; 2 Division of Hematology and Oncology, University of Florida College of Medicine, Gainesville, USA; 3 Department of Pathology, University of Florida College of Medicine, Gainesville, USA; 4 Division of Hematology and Oncology, University of Florida, Gainesville, USA

**Keywords:** t-cell lymphoma

## Abstract

Herein, we present the case of a 63-year-old female with a history of Behçet’s disease managed with long-term prednisone and azathioprine who initially presented for symptomatic anemia, which progressed to pancytopenia with neutropenic fever. Initial workup ruled out infectious etiologies but was indeterminate for immune-mediated or neoplastic causes. Bone marrow biopsy demonstrated a CD8+ gamma-delta T-cell neoplasm; however, imaging and skin biopsy pathology did not support hepatosplenic or cutaneous lymphoma involvement. By the 2017 World Health Organization (WHO) classifications, these findings were defined as gamma-delta peripheral T-cell lymphoma, not otherwise specified (NOS). This is suspected to be secondary to chronic immunosuppression from long-term steroid and azathioprine use. The patient was treated with one cycle of the EPOCH chemotherapy regimen ((etoposide, vincristine, cyclophosphamide, doxorubicin, and prednisone), but the treatment course was complicated by an angioinvasive fungal infection and the patient subsequently transitioned to symptom-focused therapy in a hospice facility.

## Introduction

Gamma-delta T-cell lymphomas (GDTCL) are a rare subset of peripheral T-cell lymphomas that make up 1%-5% of T-cell lymphomas in the general population [1-3]. Most GDTCL fit into two subtypes described by the 2017 World Health Organization (WHO) classification of lymphoid neoplasms: hepatosplenic GDTCL, with associated hepatosplenomegaly and systemic symptoms, and primary cutaneous GDTCL with characteristic panniculitis and dermatological manifestations. Both subtypes of GDTCL are clinically aggressive malignancies that are often refractory to chemotherapy, with a median survival of one to two years and 10-15% five-year overall survival [4].

The rarity of this subtype of lymphoma and its aggressive clinical course underscore the need for early diagnosis and appropriate treatment. In our case, the absence of cutaneous or hepatosplenic disease obscured the diagnosis and necessitated prompt clinical recognition and histopathological and immunophenotypical classification in order to facilitate treatment or identification of clinical trials. An interdisciplinary approach to treatment was essential. This case also emphasized how infectious sequelae of aggressive therapy and interaction with comorbid conditions can complicate a patient’s clinical course and necessitate anticipatory symptom management.

By summarizing this unique patient case, we hope to contribute to the understanding of GDTCL and its rare unspecified subtype, provide insight into its diagnostic workup, and discuss treatment options.

## Case presentation

Initial presentation

A 63-year-old female with a past medical history of Behçet’s disease initially presented to the emergency department with fatigue and dyspnea. She was diagnosed with symptomatic macrocytic anemia, and she was transfused two units of packed red blood cells. Workup included complete blood count (CBC) with peripheral smear review, reticulocyte count, serum lactose dehydrogenase level, haptoglobin level, direct Coombs test, vitamin B12 level, folic acid level, copper level, and peripheral blood flow cytometry, and it was unrevealing. Daily azathioprine for Behçet’s disease was discontinued due to concern for possible bone marrow suppression.

The patient’s Behçet’s disease was diagnosed at age 19 after she developed chronic arthralgias, oral and vaginal ulcers, and recurrent fevers. She had no history of ocular or cutaneous lesions, anemia, or transfusions. She was treated for the past 27 years with oral prednisone 16 mg daily and 1.5 mg nightly, and oral azathioprine 100 mg twice daily. Five months prior to this presentation, her azathioprine dosage was reduced to 50 mg twice daily. She had never taken biologic medications. Esophagogastroduodenoscopy and colonoscopy within the past year were normal. She also had a history of type 2 diabetes mellitus associated with chronic steroid use and hypothyroidism. She was a retired teacher with no history of smoking, intravenous drug use, or heavy alcohol use. The reported family history was significant only for breast cancer in her sister.

One month later, the patient returned to the emergency department with recurrent dyspnea and fatigue, as well as intermittent fevers, headaches, arthralgias, right ankle erythema, and intermittent confusion. She was tachycardic and tachypnic with orthostatic hypotension, mild jaundice, and scleral icterus. Labs at this time showed pancytopenia with 1% blasts and bilirubin and liver function studies within normal limits. The patient received additional red blood cell transfusions, and empiric broad-spectrum antibiotics and antifungals were initiated for neutropenic fever. Multiple blood and urine cultures were negative for an infectious cause. The peripheral blood smear showed severe leukopenia with neutropenia, moderate macrocytic anemia, mild to moderate thrombocytopenia, and rare blasts. Cerebrospinal fluid (CSF) analysis, MRI of the brain and right ankle, and CT of the chest, abdomen, and pelvis with intravenous (IV) contrast were nondiagnostic. Fever, ankle erythema, and diffuse arthralgias briefly improved with IV steroids, suggesting a possible vasculitic etiology. The initial bone marrow biopsy identified an abnormal T-cell population suspicious for CD8+ T-cell lymphoproliferative disorder by flow cytometry but had insufficient marrow present for definitive diagnosis. Due to ongoing transfusion dependence for symptomatic anemia and recurrent fevers, the patient was transferred to a tertiary care center for oncology and rheumatology consultation services.

Investigations

On transfer to a tertiary care center, the patient was pancytopenic with a white blood cell count of 100/mm^3^, hemoglobin 8.5 g/dL, hematocrit 25.4%, and platelet count 29,000/mm^3^. Blood smear showed pancytopenia with severe leukopenia, moderate macrocytic anemia, mild to moderate thrombocytopenia, absolute neutropenia, a left shift, and rare blasts. Cytomegalovirus (CMV) quantitative deoxyribonucleic (DNA) polymerase chain reaction (PCR) was 446 copies/mL with no detectable CMV immunoglobulin G (IgG) antibody. Coagulation studies, vitamin B12, and folate levels were within normal limits, and hepatitis B and C panels were negative. Documentation from the outside hospital reported normal levels of copper and negative HIV testing, but no lab results were available.

**Figure 1:**
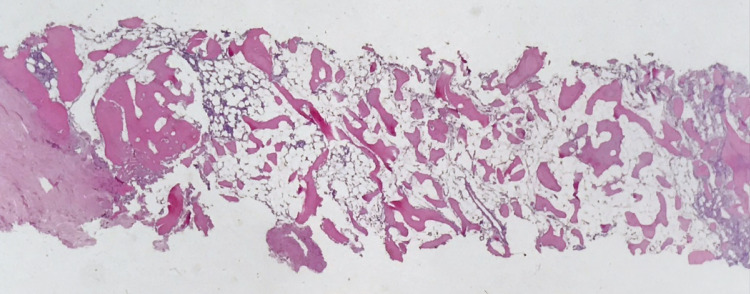
Bone marrow biopsy hypocellularity Hematoxylin and eosin, 20x magnification. The marrow is variably hypocellular for age (5%-30%).

**Figure 2:**
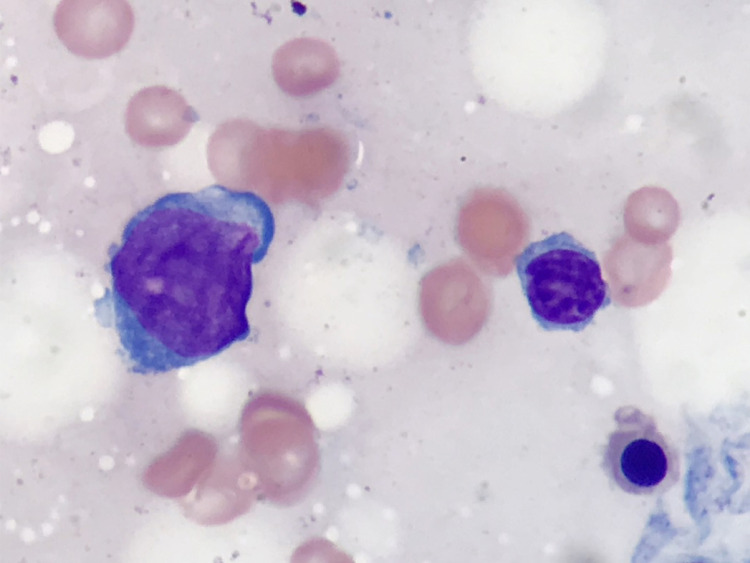
Atypical lymphocytes in bone marrow biopsy Wright-Giemsa, 1000x magnification Atypical lymphocytes (left) are present in touch preparations. These lymphocytes are large, with relatively smooth chromatin and deep blue cytoplasm, seen in comparison to a benign-appearing lymphocyte (right).

**Figure 3:**
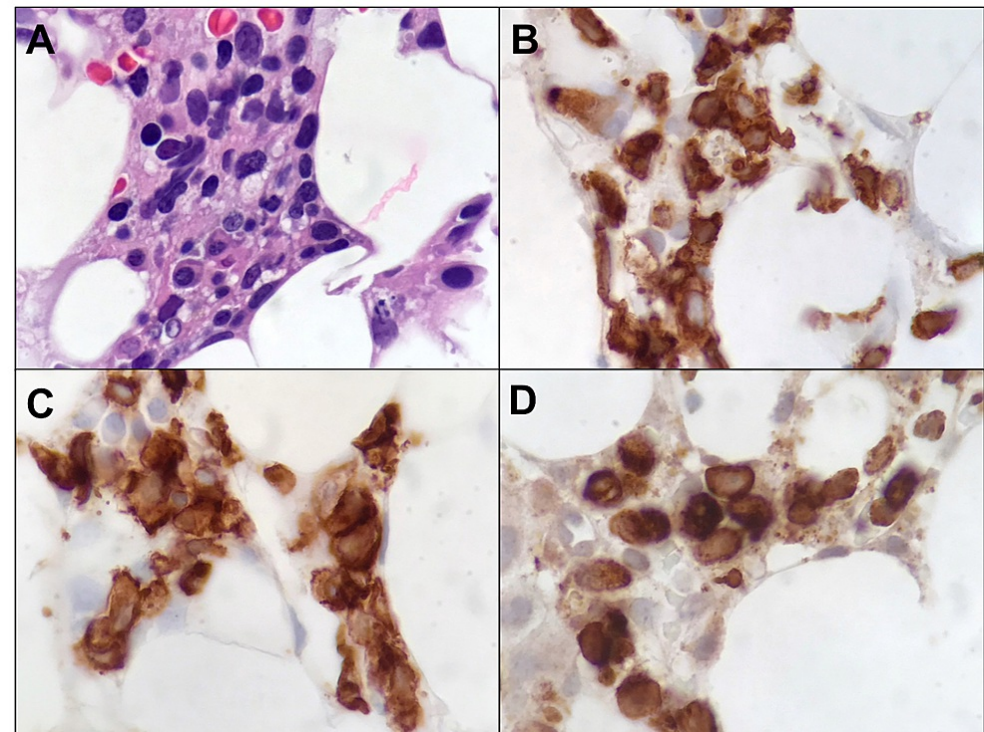
Bone marrow biopsy histopathology 1000x magnification In areas with higher marrow cellularity, a large atypical lymphoid infiltrate is noted (A, hematoxylin and eosin). These lymphocytes express CD3 (B), CD8 (C), and perforin (D) by immunohistochemical staining, consistent with a cytotoxic phenotype.

**Figure 4:**
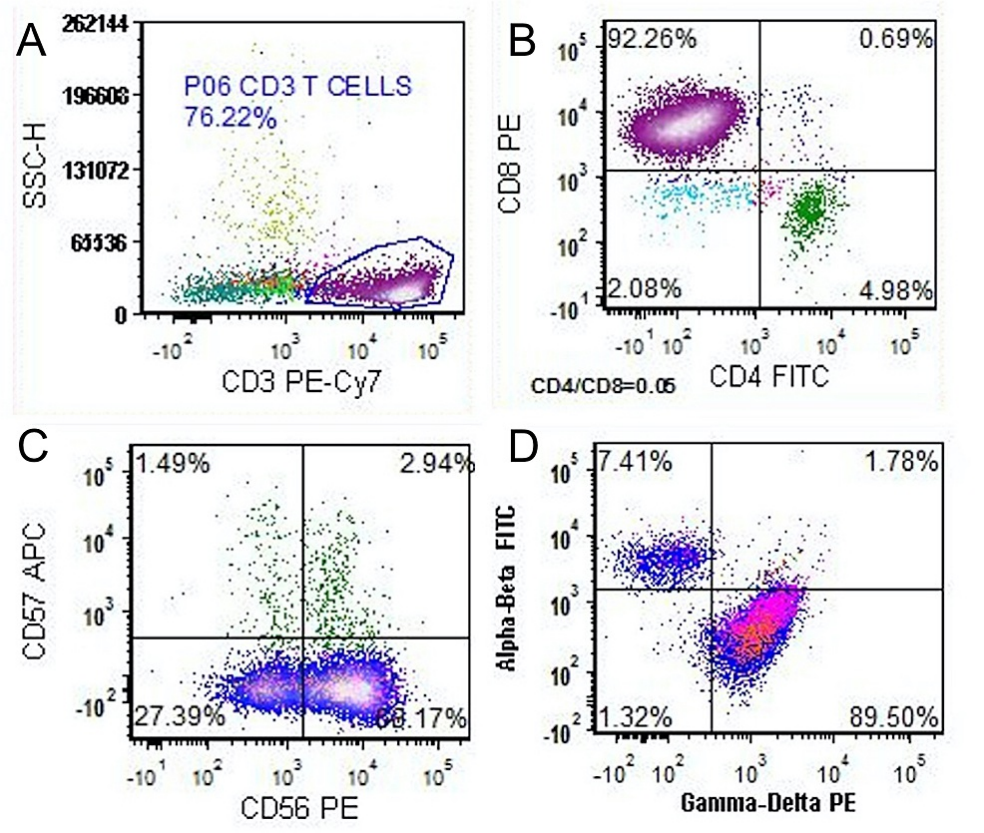
Flow cytometry of bone marrow aspirate Flow cytometric analysis performed on the bone marrow aspirate shows a preponderance of CD3-positive T cells (A), expressing CD8 (B) and CD56 (C). These T cells were of the gamma-delta subtype (D).

A repeat bone marrow biopsy showed a cytotoxic gamma-delta T-cell lymphoproliferative neoplasm (60% of marrow cells), variably hypocellular marrow (5%-30%) with reduced trilineage hematopoiesis, and relative erythroid hyperplasia and myeloid hypoplasia (Figure 1). Large abnormal lymphocytes with paucigranular cytoplasm were observed, consistent with cytotoxic large granular lymphocytes (Figure 2). The bone marrow space was extensively involved by abnormal T-cells expressing CD3, CD8, CD2, CD5, CD45, gamma/delta, CD7, CD56, CD16, and perforin, and not overtly expressing TDT and CD30 (Figure 3, panels A-D, Figure 4 panels A-D). PCR analysis of the T-cell receptor gamma gene rearrangement showed monoclonal distribution. Bone marrow also showed that 50% of CD8+ T-cells were Ki-67+, consistent with high proliferative activity.

Results of cytogenetic evaluation included:

46,XX,der(13)t(7;13)(q11.2;p11.1)

46,idem,der(10)t(8;10)(p11.2;p11.2)

45,idem,-10,add(11)(q25),der(22)t(10:22)(q11.2;p11.1)

46,XX

The patient’s evaluation for neutropenic fever included bacterial and fungal blood cultures that remained negative throughout hospitalization. Urine cultures also showed no growth throughout hospitalization. A lumbar puncture with cerebrospinal fluid (CSF) analysis showed normal cytology, protein, and glucose with no morphologic evidence of lymphoma. CSF gram stain and culture were negative. Cryptococcal antigen testing was negative.

Treatment

**Figure 5:**
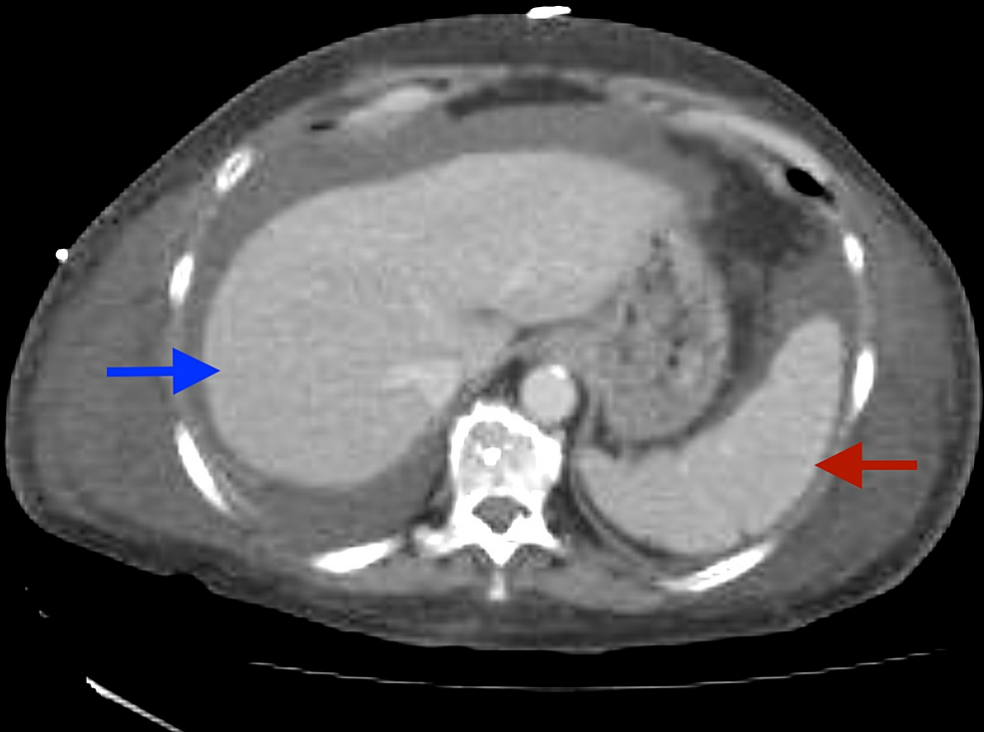
CT abdomen CT of the chest, abdomen, and pelvis performed after Cycle 1 of CHOP (cyclophosphamide, doxorubicin hydrochloride, vincristine sulfate, and prednisone) showed anasarca with ascites and pleural effusion. Notably, as seen on this representative image, the liver (blue arrow) and spleen (red arrow) appeared normal with no evidence of hepatosplenomegaly or of any extramedullary involvement of T-cell lymphoma.

Due to the aggressive nature of gamma-delta peripheral T-cell lymphomas, the EPOCH combination chemotherapy regimen (etoposide, vincristine, cyclophosphamide, doxorubicin, and prednisone) was rapidly initiated. A post-induction bone marrow biopsy on Day 23 of Cycle 1 showed less than 1% residual disease, and repeat CT of the chest, abdomen, and pelvis showed no evidence of extramedullary lymphoma involvement (Figure 5).

**Figure 6:**
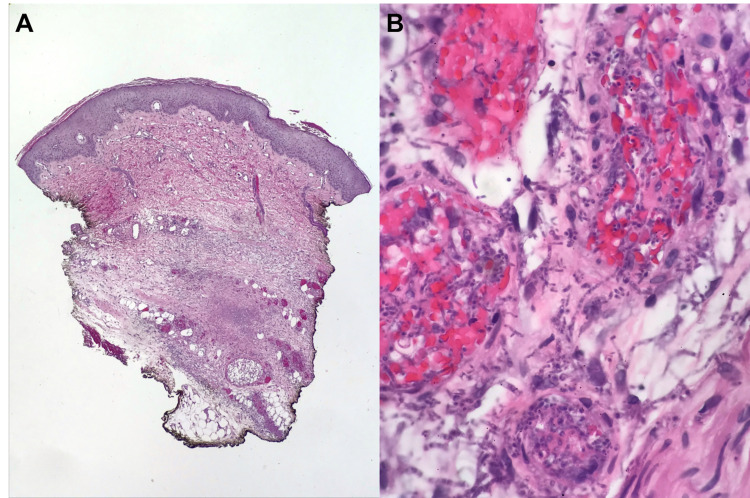
Lower extremity rash punch biopsy pathology Hematoxylin and eosin The epidermis and papillary dermis are largely unremarkable, without a significant lymphocytic infiltrate (A, 20x magnification). However, the deeper dermis (B, 600x magnification) shows invasion by fungal organisms, predominantly localized within and around the deep dermal blood vessels with associated vasculitis and thrombus formation.

On Cycle 1 Day 5, she developed an erythematous rash of the right lower extremity initially suspicious for recurrent vasculitis, which progressively developed into eschars at multiple sites. A punch biopsy was performed, which revealed yeast and hyphal elements localized within and around the deep dermal blood vessels with associated vasculitis, necrosis, and thrombus formation (Figure 6), and biopsy cultures grew Paecilomyces species. An additional biopsy of a right upper extremity rash also showed fungal organisms with yeast and hyphal forms predominantly in the deeper dermis. Cluster of differentiation 3 (CD3) and CD20 immunostains revealed only scattered T cells and were negative for B cells, with no evidence of cutaneous lymphoma involvement.

Her hospital course was further complicated by *Clostridium (C.) difficile* colitis treated with oral vancomycin, intermittent hyperglycemia due to steroid adjustment for Behçet’s disease, and left upper extremity deep vein thrombosis treated with heparin infusion.

Supportive care throughout her hospitalization included blood transfusions, filgrastim, IV fluids, physical therapy, occupational therapy, massage therapy, topical lidocaine, and IV and oral pain medications.

Outcome

On Cycle 1 Day 25, the patient developed expressive aphasia and an MRI of the brain demonstrated nonspecific patchy signal changes of the pons and midbrain without a discrete intracranial lesion. Subsequent CSF studies and flow cytometry were negative for infection or lymphoma, and her neurologic status returned to baseline over the following two days.

**Figure 7:**
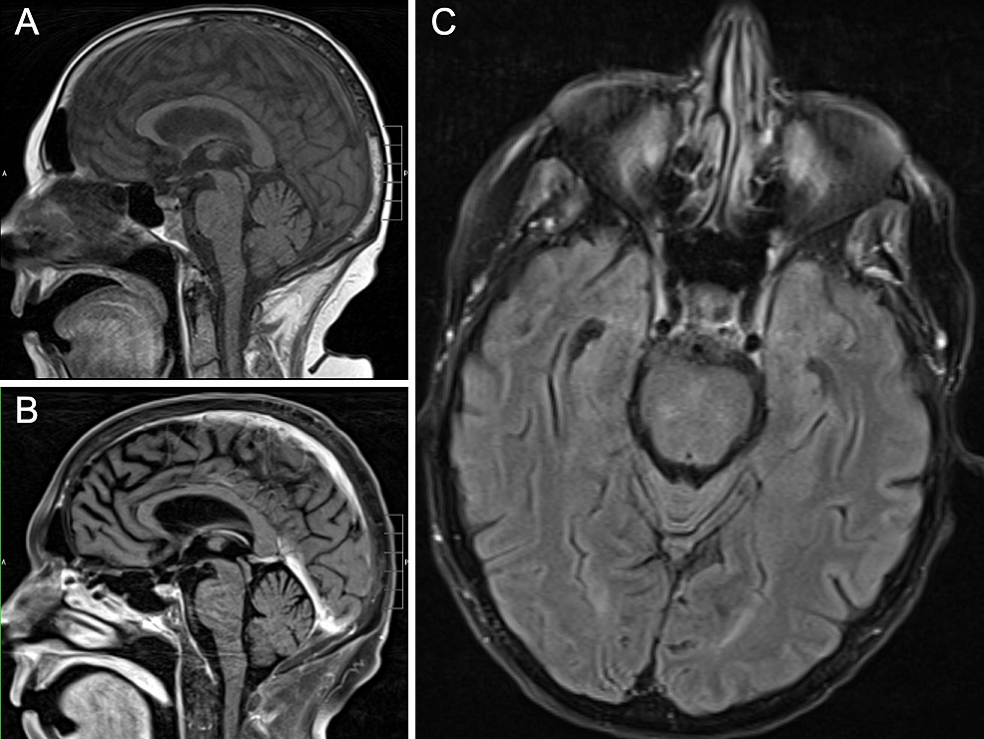
MRI brain Brain MRI was performed to evaluate encephalopathy that developed during the hospital course. Representative sagittal views (A, T1 FLAIR and B, T2 fluid-attenuated inversion recovery (FLAIR)) and transverse view (C, T2 FLAIR) showed no evidence of meningitis, encephalitis, mass lesions, hydrocephalus, acute ischemia, or hemorrhage.

Due to the complicated disease course and multiple infections, additional chemotherapy cycles were delayed. Subsequent progression of the fungal infection and other comorbidities led to a decline in performance status with recurrent encephalopathy and sepsis. On Day 33 after the first cycle of chemotherapy, the patient decided to transition to comfort-focused care and transfer to a hospice facility. The patient died four days later at the hospice facility.

## Discussion

Differential diagnosis

The primary differential diagnosis for the patient’s pancytopenia included nutritional, infectious, inflammatory, malignant, and drug-induced etiologies.

Nutritional causes, such as B12, folate, or copper deficiency, were ruled out by normal range laboratory testing. The patient had no reported history of excessive alcohol use. Peripheral destruction of cell lines was ruled out with negative Coombs’ testing and the absence of schistocytes on review of the peripheral blood smear. Prothrombin time (PT), partial thromboplastin time (PTT), and D-dimer were within normal limits, ruling out disseminated intravascular coagulation with the absence of schistocytosis. Sepsis-induced cytopenias were also considered due to her fever and tachycardia at her second presentation, but infectious workup including blood and urine cultures, cerebrospinal fluid analysis (CSF), and CT of the chest, abdomen, and pelvis revealed no infectious causes.

At the time of her initial presentation, azathioprine-induced bone marrow suppression was strongly considered; however, pancytopenia worsened even after discontinuation of azathioprine. An erythematous and tender rash of her right lower extremity and the improvement of her symptoms with IV methylprednisolone at the outside hospital alternatively suggested an inflammatory or vasculitic etiology. Diagnoses considered included a flare of her Behçet’s disease, polyarteritis nodosa, or cutaneous small-vessel vasculitis. Notably, this rash self-resolved prior to treatment and did not fit the pattern of cutaneous T-cell lymphoma.

Malignant etiologies were also high on the list of differential diagnoses due to the patient’s long history of immunosuppression and the initial bone marrow biopsy suspicious for CD8+ T-cell lymphoproliferative disorder. The patient’s lower extremity panniculitis-like skin lesions that appeared after chemotherapy raised suspicion for cutaneous lymphomas, such as mycosis fungoides and subcutaneous panniculitis-like T-cell lymphoma, but these were effectively ruled out by biopsy of multiple skin lesions consistently showing no evidence of lymphoma. Peripheral T-cell lymphoma was also considered, and in the absence of distinct cytogenetics or hematopathology, peripheral T-cell lymphoma became the working diagnosis.

A repeat bone marrow confirmed a CD8+ gamma-delta T-cell lymphoma, however, we could not establish a diagnosis of any existing subtype. The primary cutaneous subtype of GDTCL was unlikely, as biopsies of the patient’s areas of rash and necrosis showed no evidence of cutaneous lymphoma. Hepatosplenic GDTCL was also unlikely, as CT imaging showed no hepatosplenomegaly. Additionally, the patient’s bone marrow cytogenetic analysis did not show isochromosome 7q, which is strongly associated with hepatosplenic GDTCL. Due to the patient’s clinical status, a diagnostic hepatic core needle biopsy was deferred. A subset of T-cell large granular lymphocyte leukemia can present with a gamma-delta phenotype, but this diagnosis was unlikely due to the absence of characteristic large intracytoplasmic granules on pathological evaluation. After several interdisciplinary discussions including a review of the case with pathology, the final diagnosis was determined to be gamma-delta T-cell lymphoma, not otherwise specified.

Gamma-delta T-cells are lymphocytes of thymic origin with gamma and delta chains making up the T-cell receptor heterodimer. These lymphocytes play a role in the innate immune response. Gamma-delta T-cells make up less than 5% of peripheral T-cells and show tropism for various tissues, including the red pulp of the spleen, lymph nodes, and gastrointestinal and skin epithelia. GDTCLs are a rare subset of PTCLs that are clinically aggressive with poor response to treatment and poor long-term survival [1-3].

Hepatosplenic GDTCL are associated with chronic immunosuppression in patients with solid organ transplants or autoimmune conditions such as Crohn’s disease treated with purine analogs or TNF inhibitors [5-6]. This patient’s chronic azathioprine use for over 20 years suggested that she was at higher risk for this GDTCL subtype; however, she lacked the pathognomonic hepatosplenic involvement. Adding to the diagnostic dilemma was the similarity in the clinical appearance of her angioinvasive fungal infection to the panniculitis-like nodular plaques seen in cutaneous GDTCL, which generally occur in older patients (median age 61) but lack an association with chronic immunosuppression [7-8]. This case could not be attributed to any of the existing subtypes of GDTCL and adds to the growing published collection of rare cases of unspecified GDTCLs of the lymph nodes, intestinal epithelium, central nervous system, and bone marrow [4,9-15]. These cases indicate a spectrum of clinical presentations of GDTCL that do not fit neatly into the current classification system.

Due to the rarity of GDTCL, there are no existing guidelines or current targeted clinical trials investigating management and outcomes; however, some cases have been included in clinical trials of patients with PTCLs and evaluated in a subset analysis. One study reported that anthracycline-containing chemotherapy regimens, such as CHOP and Hyper-CVAD (cyclophosphamide, vincristine, doxorubicin hydrochloride, dexamethasone, cytarabine, mesa, methotrexate, and leucovorin), are commonly used with 30%-45% response rates and a median time to relapse of four months, and some success with either autologous or allogenic hematopoietic stem cell transplantation [1]. While brentuximab-vedotin is now part of the standard therapy for CD30-positive T-cell lymphomas and has been effective in CD30-positive cutaneous GDTCL, it was not a therapeutic option in this case due to CD30 negativity [16-17].

Another study reviewed data from 1429 PTCL cases collected as part of the T-cell Project, an international, prospective T-cell lymphoma registry. This database included 99 patients with GDTCL and anthracycline-based regimens were used in 88% of cases. Overall response rates (ORR) for chemotherapy were 45%, with 31% achieving complete remission (CR). There were lower CR rates in peripheral GDTCL and poor response in all groups to high-dose rescue therapies [18]. There have also been 11 individual cases published of GDTCL not otherwise specified (NOS), which are summarized in Table 1.

**Table 1:** Summary of published cases of GDTCL NOS CR: complete remission; CHOP: cyclophosphamide, doxorubicin hydrochloride, vincristine sulfate, and prednisone; LSG9: vincristine, cyclophosphamide, doxorubicin, bleomycin, etoposide, methotrexate, procarbazine; ESHAP: etoposide, methylprednisolone, AraC, cisplatin; ABMT: vincristine, nimustine, etoposide, prednisolone; EPOCH: etoposide, vincristine, cyclophosphamide, doxorubicin, and prednisone; VEPA: vincristine, cyclophosphamide, doxorubicin, prednisone; MACOP-B: methotrexate, leucorvin, bleomycin, doxorubicin, cyclophosphamide, vincristine, dexamethasone; GDTCL: gamma-delta T-cell lymphomas; NOS: not otherwise specified

Patient Characteristics	Primary disease site	Treatment	Outcome
Female, 36 [9]	Lung	Chemotherapy (unspecified)	Alive and in remission at 8 months
Female, 68 [9]	Orbit	Excision only	Alive in remission at 48 months
Female, 57 [9]	Lymph node	Chemotherapy (unspecified)	Alive in remission at 10 months
Male, 29 [9]	Tongue	Excision only	Alive in remission at 15 months
Male, 29 [9]	Lymph node	Chemotherapy (unspecified)	Died of disease after 5 months
Female, 74 [4]	Bone Marrow	Eight cycles of CHOP	Died of disease after 16 months
Female, 70 [10]	Lymph node	Unspecified course of vincristine, vindesine, cyclophosphamide, doxorubicin, etoposide, procarbazine, and prednisolone	CR for 13 months, relapsed and died of disease after 28 months
Male, 64 [11]	Lymph node	One course LSG9, 50 Gy irradiation	CR for 2 months, relapse and died of disease after 9 months
Male, 66 [11]	Lymph node	Five cycles of CHOP, one cycle of ESHAP, one cycle of ABMT salvage, 11 Gy irradiation	No response, died of disease after 5 months
Male, 79 [11]	Lymph node	One cycle of CHOP, two cycles of EPOCH	No response, died of disease after 3 months
Male, 72 [12]	Lymph node	One cycle of VEPA, one cycle of MACOP-B, 50 Gy irradiation, and one cycle of vincristine, cyclophosphamide, farmorubicin, cymerin, and prednisone salvage	CR for 1 month, second CR for 2 months, relapsed and died of disease (fever, renal failure) after 24 months

The limited understanding of the pathophysiology of GDTCL, paucity of effective evidence-based treatments or clinical trials for existing subtypes of gamma-delta T-cell lymphomas, and the difficulty in updating existing classifications underscore the challenges to treating these rare cancers.

## Conclusions

This case adds to the growing number of cases of gamma-delta T-cell lymphomas that do not fit into the existing World Health Organization subtypes, emphasizing a need for additional clinical, pathological, and cytogenetic characterization of this group of lymphomas. The association of long-term immunosuppression with unclassified subtypes of gamma-delta T-cell lymphoma may also indicate a possible characterized risk factor for these malignancies.

The aggressive course and lack of effective treatment options for gamma-delta T-cell lymphomas underscore the need for rapid recognition and enrollment in clinical trials in order to investigate optimal treatment strategies. Early recognition and involvement of multidisciplinary care, including infection control, is essential for treatment.
